# Achieving global malaria eradication in changing landscapes

**DOI:** 10.1186/s12936-021-03599-0

**Published:** 2021-02-02

**Authors:** Kimberly M. Fornace, Adriana V. Diaz, Jo Lines, Chris J. Drakeley

**Affiliations:** 1 Faculty of Infectious and Tropical Diseases, London School of Hygiene and Tropical Medicine, London, UK; 2 Centre for Climate Change and Planetary Health, London School of Hygiene and Tropical Medicine, London, UK; 3 Pathology and Population Sciences, Royal Veterinary College, Hatfield, UK

## Abstract

Land use and land cover changes, such as deforestation, agricultural expansion and urbanization, are one of the largest anthropogenic environmental changes globally. Recent initiatives to evaluate the feasibility of malaria eradication have highlighted impacts of landscape changes on malaria transmission and the potential of these changes to undermine malaria control and elimination efforts. Multisectoral approaches are needed to detect and minimize negative impacts of land use and land cover changes on malaria transmission while supporting development aiding malaria control, elimination and ultimately eradication. Pathways through which land use and land cover changes disrupt social and ecological systems to increase or decrease malaria risks are outlined, identifying priorities and opportunities for a global malaria eradication campaign. The impacts of land use and land cover changes on malaria transmission are complex and highly context-specific, with effects changing over time and space. Landscape changes are only one element of a complex development process with wider economic and social dimensions affecting human health and wellbeing. While deforestation and other landscape changes threaten to undermine malaria control efforts and have driven the emergence of zoonotic malaria, most of the malaria elimination successes have been underpinned by agricultural development and land management. Malaria eradication is not feasible without addressing these changing risks while, conversely, consideration of malaria impacts in land management decisions has the potential to significantly accelerate progress towards eradication. Multisectoral cooperation and approaches to linking malaria control and environmental science, such as conducting locally relevant ecological monitoring, integrating landscape data into malaria surveillance systems and designing environmental management strategies to reduce malaria burdens, are essential to achieve malaria eradication.

## Background

Malaria continues to be a major public health burden globally, with over 200 million cases in 2018. Despite effective treatment and control measures, over 400,000 deaths are caused by malaria annually, primarily in sub-Saharan Africa [1]. Malaria eradication, the permanent reduction of malaria infections globally to zero, has been a long-standing goal of the public health community, with a previous failed malaria eradication attempt from 1955–1969 [2]. Following significant reductions in malaria morbidity and mortality between 2000 and 2015, the World Health Assembly endorsed aims to reduce malaria burdens a further 90% by 2030 and has again begun exploring the possibility of malaria eradication [3]. Within the past year, two separate initiatives, the World Health Organization (WHO) Strategic Advisory Group for Malaria Eradication (SAGme) and the Lancet Commission on Malaria Eradication analysed future scenarios, concluding that malaria eradication is feasible and outlining key priorities [4, 5]. Both reports examine the impacts of global environmental change and conclude long-term climate patterns and urbanization are likely to be favourable for malaria eradication. Within these assessments, land use and land cover changes (LULCC) are only recognized as external factors influencing malaria transmission and not as a priority for eradication campaigns due to the difficulty predicting impacts.

LULCC, such as deforestation, agricultural expansion and infrastructure development, have huge potential to impact malaria control efforts through disruptions of both ecological and social systems [6]. Natural geographic heterogeneity in malaria is largely driven by biological differences in *Anopheles* species adapted to different landscapes while human vulnerability, economic status and access to healthcare are intricately linked with local environmental factors. The efficacy of malaria interventions and vector control measures are largely dependent on these factors and a successful malaria eradication campaign needs to develop landscape-specific strategies. Within countries moving towards elimination, many remaining foci of malaria transmission are driven by landscape factors, such as the high malaria incidence associated with deforestation in Southeast Asia and South America [7]. Conversely, many major malaria elimination successes were underpinned by LULCC, including, famously, the extensive hydrological and agricultural modifications conducted by Italian malaria control programmes following World War II [8]. Because LULCC are dynamic processes, impacts on transmission change over time following initial environmental changes and subsequent development. Anthropogenic changes generally reduce biodiversity, favouring species adapted to human populations. As land is transformed at unprecedented rates, there is a danger that future development will embed malaria into these landscapes, creating ideal man-made habitats for *Anopheles* vectors. Alternatively, the expected extent of future development offers unparalleled opportunities to “build out” malaria, reducing background transmission sufficiently to enable malaria eradication.

In this article, based on a report commissioned by the SAGme, a framework is outlined for incorporating LULCC into malaria eradication strategies. While previous successful disease eradication programmes for smallpox and rinderpest relied heavily on vaccination, there remains no highly effective licensed vaccine for malaria and increasing levels of insecticide resistance threaten to undermine existing vector control methods [9]. Emergence of zoonotic malaria in Southeast and South America presents new challenges for eradication and requires explicit consideration of LULCC on wildlife habitats. Within this context, it is clear a successful malaria eradication strategy will need to both mitigate the negative impacts of LULCC and leverage LULCC beneficial to malaria control. Effective strategies are inherently interdisciplinary and cannot be implemented solely within health sectors, requiring engagement of agricultural scientists, engineers, geographers and other disciplines to monitor and mitigate impacts of LULCC on malaria transmission [10]. Although interactions between human and natural systems driving malaria transmission are undoubtedly complex, this should not preclude explicit consideration of LULCC into eradication strategies. This article outlines the extent and drivers of LULCC, review the evidence on direct and indirect impacts on malaria transmission and identify priorities for malaria control and eradication, using landscape data to inform malaria surveillance and control while in turn incorporating malaria risks into land management strategies.

## Land use and land cover changes: definitions and drivers

Land cover refers to the physical and biological cover of terrestrial surfaces, such as water, soil, vegetation and infrastructure, while land use refers to the human management and activities which modify land surface processes [11]. Although people have transformed landscapes since prehistoric times, the extensive changes in the past 300 years following the Industrial Revolution have been unprecedented, leading to this era being termed the Anthropocene [12]. While agricultural land occupied less than 2% of global ice-free land prior to 1000 AD, this percentage increased to over 4% in 1700 AD to 35% in 2000 AD [13]. Today, over 75% of Earth’s ice-free land has been altered by human residence and land use [14].

**Fig. 1:**
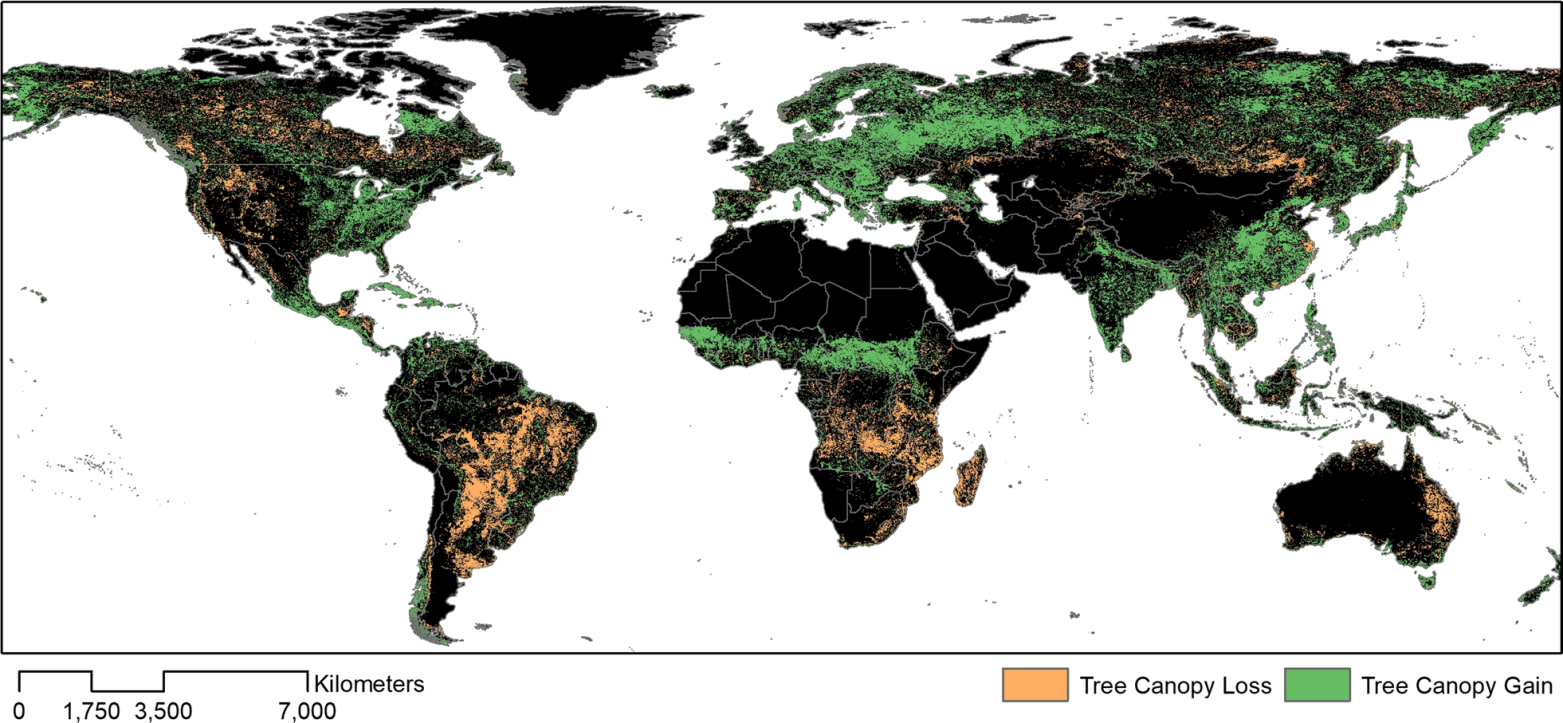
Net forest canopy cover loss and gain between 1982 and 2016 [115]

Deforestation remains one of the main global LULCC (Fig. 1). Changes to forest cover are particularly pronounced in tropical areas, where over 80% of new agricultural land was cleared from tropical rainforests between 1980 and 2000 and an estimated 2100 km^2^ of forests were lost per year between 2000 and 2012 [15, 16]. Much of this deforestation is driven by agricultural expansion driven by rising demands due to population growth and increased consumption levels [17]. Between 1970 and 2010, there has been a 1.4-fold increase in the number of livestock and an 18.4% increase in daily per capita food availability globally [18]. However, increased productivity and industrialization has meant this increase in the amount of food produced is not always accompanied by corresponding increases in land area but rather new management techniques, such as irrigation and fertilizers [19]. For example, there was a 73% increase in the area of irrigated land between 1970 and 2010 [20]. Global biofuel production is also increasing rapidly, growing 19.4% per year globally between 2004 and 2011, with an expansion of 33.2 million hectares for oilseeds globally [21].

The causes for LULCC are multifactorial, with underlying political, institutional and economic factors driving agricultural expansion, resource extraction and infrastructure development [22]. For example, while extensive deforestation occurred in Indonesia between 2000 and 2012, commitments to global climate change agreements led to substantial decreases in forest loss in 2017 [23–25]. Conversely, policies may have unintended implications. Peace agreements between the Colombian government and armed groups led to land colonization in previously inaccessible areas of the Andean-Amazonian foothills of Colombia; deforestation has been further amplified by governmental programs building roads and fostering extractive and ranching industries [26]. United States drug policies have led to “narco-deforestation”, extensive forest loss in Central America fuelled by the development of landing strips, need to launder money and influxes of cash from the global narcotics trade [27]. These complex economic and social forces driving LULCC may have unintended consequences for malaria transmission, disrupting both ecological and human systems (Table 1).

**Table 1:** Examples of effects of land use change on potential malaria risks

Environmental changes	References
Deforestation	
Increases in anopheline larval breeding sites in response to forest clearing in the Amazon	[30]
Initial decreases in vector densities followed by colonization by more efficient malaria vectors	[7, 35]
Changes in vector habitat suitability linked with forest disturbance	[29, 34]
Changes in ecological structure and biodiversity increasing or decreasing vector densities, availability of blood meals and resulting disease risks	[116–118]
Agricultural expansion	
Effects of irrigation systems	[40, 119]
Expansion of rubber and rice paddies associated with increases in anopheline densities	[28, 36]
Socio-demographic changes	
Population at risk	
Influx of susceptible populations into endemic areas in response to increased economic opportunity	[43, 120]
Increase and movement of migrant worker populations in the Amazon and Southeast Asia	[121, 122]
Occupational changes, such as forestry and extraction activities bringing people into vector habitats	[44, 47]
Socioeconomic status	
Increased income following agricultural development leading to decrease in malaria risk	[52]
Improved housing structure due to development reducing malaria risks	[51, 123]
Wildlife reservoirs	
Origin of malaria	
* P. falciparum* originated from non-human primates	[54]
Spatial overlap with wildlife hosts	
Increased contact between people and non-human primates hypothesised as main driver of human infections with *P. knowlesi* and *P. cynomolgi* in Asia and *P. simium* and *P. brasilianum* in South America	[76, 85, 124, 125]
Maintenance of malaria infections	
Human malaria species circulating in great apes and gorillas in West and Central Africa	[55, 56]

## LULCC impacts on malaria transmission

Impacts on malaria transmission are complex and highly context-specific, with environmental and demographic changes within a specific setting either increasing or decreasing risks. Natural geographical variation is largely driven by biological differences between local *Anopheles* species and the landscapes to which they are adapted. LULCC changes affect these disease systems in different ways in different regions. For example, when a landscape becomes urbanized, the original natural streams and ponds are typically either drained, enclosed in concrete, or polluted with decaying organic matter. These transformations make the water unsuitable as a breeding site for all-but-one *Anopheles* malaria vector species (though other mosquitoes such as *Culex quinquefasciatus* can thrive). For this reason, there is often little or no transmission in the thoroughly urbanized centres of large African cities, despite intense transmission in the surrounding countryside. In India, by contrast, there is *Anopheles stephensi*. the world’s only important *Anopheles* species that is well-adapted to urban conditions, through its ability to breed in man-made containers, including domestic water storage containers. Because of these differences in vector species, urbanization has different impacts on malaria geographically.

Anthropogenic LULCC is one element of a complex development process with economic, agricultural and social dimensions. As these components all affect malaria and occur simultaneously, it is difficult to distinguish between effects of landscape, housing, health coverage and other factors. In north-western Europe, malaria gradually disappeared between 1550 and 1950, not due to public health interventions, but from cumulative shifts in land use, including drainage of marshes, shifts in animal husbandry, and improvements in housing. Similarly, the introduction of house-spraying and improved drugs in the mid-twentieth century enabled elimination to be achieved in Southern Europe, the USA and several Caribbean islands. However, environmental, economic and social factors were equally important, reducing background transmission to the point where elimination was within reach and making malaria absence a stable state after the withdrawal of anti-malaria spraying despite the re-introduction of infection by imported cases. This section outlines how LULCC impacts vector, human and wildlife systems, highlighting the linkages between these.

## Impacts on vector biology

LULCC directly affects anopheline mosquito populations, altering the abundance, species composition and life history of malaria vectors. Ecological changes in soil, sunlight cover, vegetation type, development of water pockets and water temperature, affect breeding conditions for *Anopheles* malaria vectors with effects varying by *Anopheles* species. For example, while deforestation reduces shaded water bodies, the preferred breeding habitats of some *Anopheles* species, other *Anopheles* species thrive in water bodies with increased sunlight, with increased larval survivorship, adult productivity, intrinsic growth rates and shortened gonotrophic cycles significantly increasing vectoral capacity [28]. Other environmental and microclimate changes due to LULCC may favour survival of different *Anopheles* species enabling sustained seasonal malaria transmission or impacting the availability of hosts and blood meals.

Associations between forest disturbance and vector ecology are widely described in Southeast Asia and South America. Many highly efficient forest vector species occur within these regions, breeding in forest fringe and deforested areas. For example, within Malaysian Borneo, *Anopheles balabacensis* biting rates were greater in modified forest than in primary forest, with breeding sites found in wheel tracks in logged areas [29]. Similarly, deforestation within the Amazon also resulted in increased larval breeding sites and corresponding increases in malaria incidence [30]. In the Peruvian Amazon, extensive deforestation between 1983 and 1995 undermined previous achievements of malaria eradication programmes and corresponded with a fourfold increase in malaria cases nationally between 1992 to 1997 and a 50-fold increase within the rapidly deforested Loreto Department [31]. This malaria emergence paralleled increases in *Anopheles darlingi*, which was not found in the area in 1991 and favours ecologically altered habitats, leading to increased vector density in areas undergoing rapid land use change in close proximity to human settlements [31].

However, in some sites, forest disturbance may reduce malaria risks. For example, in African sites where the deep forest species *Anopheles nili* is the main vector, deforestation leads to modest reductions in malaria transmission [7]. Alternatively, in other African sites, deforestation can create habitats for non-forest efficient vectors. In Nigeria, forest loss was demonstrated to have a large impact on malaria risks, with each standard deviation of forest loss corresponding to an almost 5% increase in malaria in children under 5 [32]. A study in the Democratic Republic of Congo similarly found deforestation and agricultural expansion led to an increase in malaria prevalence in children; these LULCC were associated with increases of indoor biting rates of the malaria vector *Anopheles gambiae *sensu lato [33].

Forest disturbance can also impact species composition and may initially deplete deep forest vectors but subsequently lead to invasion by other efficient vectors [7]. Counter-intuitively, the abundance of both colonist (disturbance-tolerant) and climax (disturbance-intolerant) anopheline mosquitoes species increased in disturbed forests in Panama [34]. *Anopheles albimanus*, a colonist species, co-existed at the landscape scale with two climax species, *Anopheles oswaldoi* and *Anopheles triannulatus*. The likelihood of colonist-vector species occurrence was most prominent at highly disturbed forest sites and decreased markedly in relatively undisturbed forest [34]. Similarly, a study in highly fragmented forested areas of Cambodia suggested decreases in primary malaria vectors but increases in secondary vectors, with the outdoor and early biting behaviours of these secondary vector species sufficient to maintain malaria transmission [35]. These impacts on species composition influence contact rates with hosts and pathogen transmission, with colonist species often more likely to transmit pathogens than climax species.

Agriculture has also been associated with changes in *Anopheles* densities due to factors such as planted crops, irrigation, applications of pesticides or changes in host availability. Rubber plantations, containing planted trees with high humidity and lower temperatures, can provide ideal environments for malaria vectors. Since the first accounts in Malaysia, regular malaria outbreaks have been reported across Southeast Asian rubber plantations [36]. As 90% of the global demand in rubber is met by the expansion of rubber plantations in Southeast Asia, with an expanding migrant workforce, malaria control in this region might be jeopardized by the rubber boom [36]. Introduction of new crop species or farming practices can also alter vector species composition. In Thailand in the 1970s, development of cassava and sugarcane plantations led to increases in malaria risks. While these agricultural changes decreased the density of the shade-loving species *Anopheles dirus*, the modified landscape provided ideal breeding conditions for the sun-loving *Anopheles minimus* and resulted in an increase in malaria transmission among resettled cultivators [28]. Other agricultural methods such as slash-and-burn techniques similarly lead to deep shade elimination, changes in the acidity and chemical composition of the soil, creation of new breeding sites in the forest fringes and higher host exposure [7]. However, while much of the literature focuses on agricultural practices driving malaria transmission, agricultural practices also can reduce transmission; for example, agroforestry is increasingly proposed as a malaria intervention in Africa where planting trees can both increase biodiversity and decrease breeding sites for sun-loving *Anopheles* vectors [37].

Irrigated rice cultivation can also create permanent habitats for mosquito larvae [38]. For example, prolongation of the breeding season of *Anopheles aconitus* caused by rice cultivation and its linked irrigation systems in Indonesia resulted in an increase of malaria incidence [28]. In sub-Saharan Africa, initiatives to systematically increase irrigated rice cultivation have resulted in a rise in prevalence of the malaria vector, *Anopheles arabiensis*. Agronomic practices, such as fertilizer and insecticide use, can increase available nutrients and create predator-free habitats, increasing larval density; conversely, use of pesticides against agricultural pests may also decrease mosquito populations. Additionally, gravid *An. arabiensis* are attracted to the odour of rice, acting as a cue for oviposition site selection [38]. However, the impacts of increased vector densities in agricultural settings on malaria transmission is unclear. Described as “paddy’s paradox,” in many cases, increased abundance may correlate with changes in biting patterns or life history or be counteracted by the socioeconomic and public health improvements associated with agriculture [39].

**Fig. 2:**
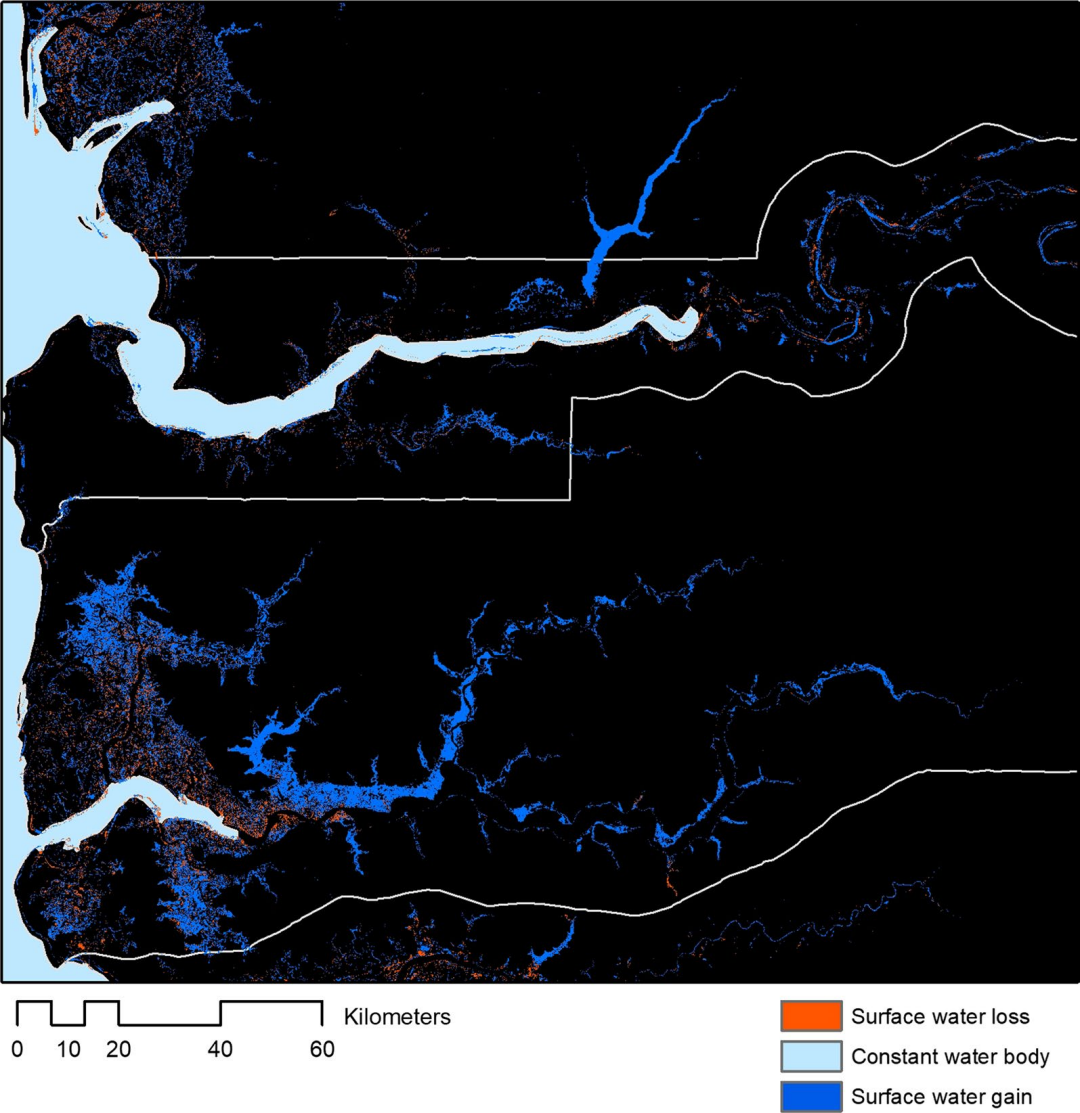
Examples of changes to surface water between 1984–2019 in The Gambia and Senegal

Wider developments of irrigation and water projects can also drive changes in vector ecology through mechanisms such as increased breeding sites, changes in water pH, turbidity and chemical composition [40]. Globally, since 1984, net increases in surface water was detected on all continents except Oceania, largely driven by reservoir creation. However, within these global trends, there are substantial fine-scale variations in changes in surface water levels and highly concentrated patterns of loss (Fig. 2) [41]. Within sub-Saharan Africa, large dams have major malaria impacts in areas of unstable transmission, either by intensifying transmission or through shifting from seasonal to perennial patterns [40, 42]. Existing large dams were predicted to increase the risk of malaria for around 15 million people, adding more than 1 million cases annually to the malaria burden in the region, with an additional 50,000 cases per year resulting from planned dams.

## Changing human populations

These ecological changes are intricately linked with the distribution, movement and quality of life of human populations. LULCC can result in influxes of immunologically naïve populations to undertake land conversion activities. This has been well described in the Brazilian Amazon, where policies encouraging development of the Amazon in the 1970s were linked to the explosive increase in malaria cases, from a total of 8,000 cases prior to the explicit government policy to up to 615,000 in the year 2000, with 99% of all malaria cases after 1990 reported in the Brazilian Amazon [43]. Termed “frontier malaria,” early stages of forest clearance are linked with changes in human exposure risks, weakened health systems and creation of vector breeding sites [44]. Risks of malaria are often highest during the initial stages of land clearing and settlement, decreasing with urbanization, agricultural expansion and increased socioeconomic status [45]. These frontier communities are often characterized by weak social institutions, limited health care and absence of malaria control measures [46].

Beyond mosquito ranges, malaria can be imported by human movements. Within the Brazilian Amazon, proximity and mobility between frontier settlements and activities explain malaria diffusion regionally [43]. Similarly, in the village of Cacao, French Guiana, a recently built road connecting the village with Brazil may have facilitated the movement of carriers from endemic areas [47]. On a national scale, analysis of mobile phone data across Kenya highlighted the role of human mobility in malaria transmission; these movement patterns are largely driven by trade and connectivity of different land use types [48].

**Fig. 3:**
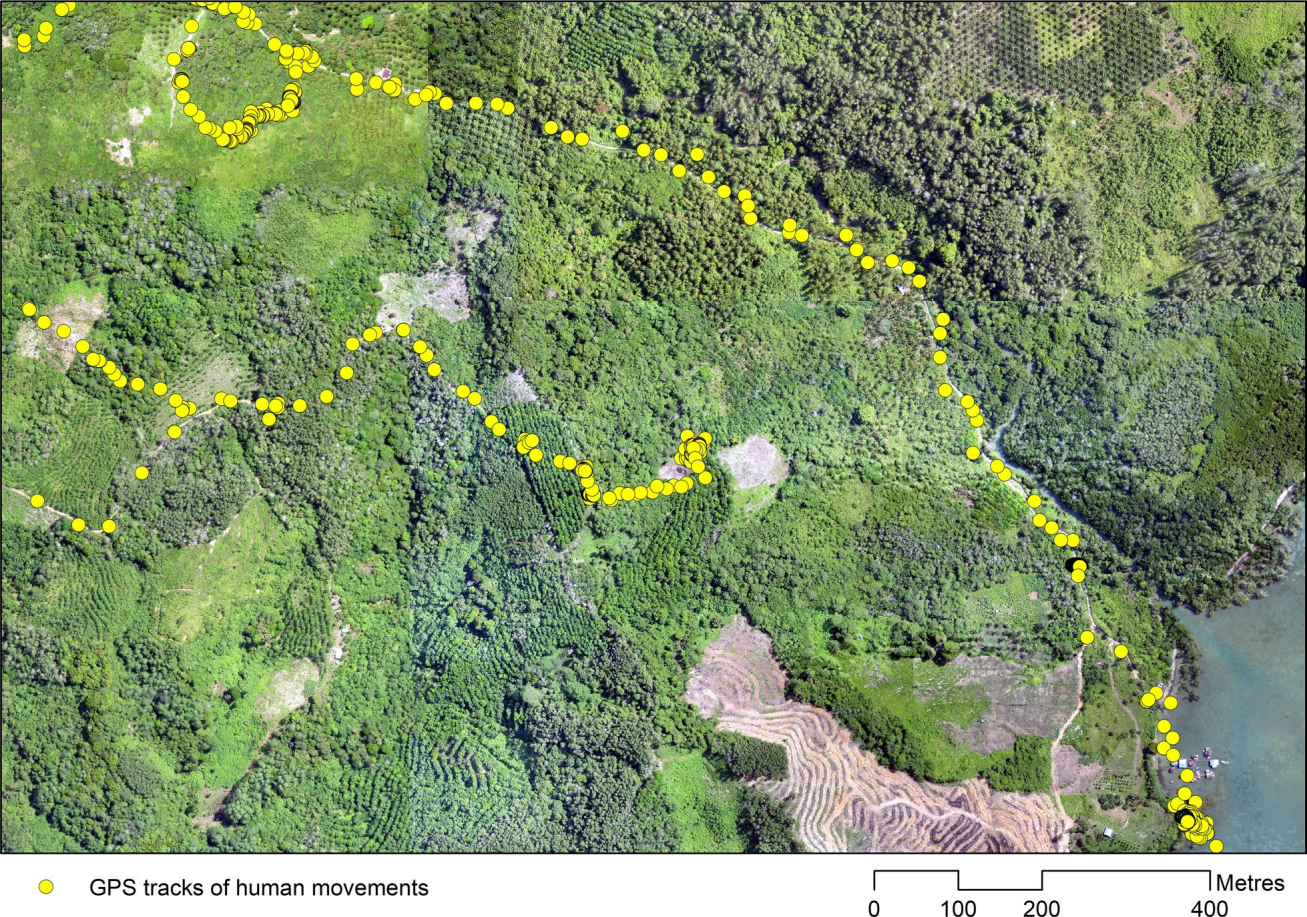
GPS tracking data showing movements of plantation worker through different vector habitats while undertaking occupational activities in Malaysian Borneo

LULCC is also accompanied by changes in specific risk behaviours and occupations as individuals undertake land conversion and agricultural activities. For example, disturbance of forest to increase farming surface has attracted seasonal workers into vector habitats in French Guiana. Risk behaviours among this migrant worker population such as outside kitchens, agricultural work during peak biting times and the absence of repellents or mosquito net use explained the spatial heterogeneity of malaria occurrence in this site [47]. Similar risk behaviours are seen among small scale gold miners in Brazil, with high population mobility facilitating parasite diffusion [43]. Within Southeast Asia, migrant workers and forest and plantation activities have similarly been identified as risk factors for malaria exposure (Fig. 3) [35, 49].

Conversely, primarily driven by economic factors, LULCC can have correspondingly positive influences on human health. In many places, initial environmental changes are followed by increases in socioeconomic status and improvements in infrastructure and public health services. For example, expansion of irrigation systems in an arid region of India was associated with dramatic increases in malaria risks; however, over time, the economic prosperity from these developments and increased health service availability led to decreased malaria incidence [50]. Modelled impacts of deforestation in frontier regions including socioeconomic factors similarly predict initial increases in malaria transmission followed by decreases due to improved socioeconomic status [45]. Economic development can improve housing quality and infrastructure, factors associated with decreasing risks of malaria [51, 52]. While differing time scales may make untangling environmental and societal impacts on malaria transmission challenging, fully understanding risks of landscape changes requires assessing how these coupled human-environmental systems interact.

## Wildlife reservoirs

LULCC impacts on vector and human populations may be further amplified by wildlife malaria reservoirs. Although four main human malarias (*Plasmodium falciparum*, *Plasmodium malariae*, *Plasmodium ovale* and *Plasmodium vivax*) are widely recognized, zoonotic malaria species such as *Plasmodium knowlesi* and *Plasmodium simium* are emerging public health threats [53]. Genetic studies suggest that human malarias such as *P. falciparum* originated from great ape species and these human malarias continue to circulate in great ape and gorilla populations in West and Central Africa [54–56]. These close evolutionary relationships, coupled with increased spatial overlap between human and non-human primate populations, present future challenges to malaria eradication.

Dramatic increases in human *Plasmodium knowlesi* cases threaten to undermine progress towards malaria elimination in Southeast Asia. *Plasmodium knowlesi* is a malaria species maintained by long and pig-tailed macaques (*Macaca fascicularis* and *Macaca nemestrina*) and transmitted by the *Anopheles leucosphyrus* group of mosquitoes [57, 58]. Since the identification of a cluster of human *P. knowlesi* infections in Malaysian Borneo in 2004, sporadic cases have been reported across Southeast Asia and *P. knowlesi* is now the main cause of human malaria in Malaysia [59–75]. Recent molecular studies have additionally identified human infections with *Plasmodium cynomolgi*, another primate malaria species carried by macaques [76–78]. LULCC, resulting in increased spatial overlap between people, macaques and mosquitoes, likely drive this emergence [58, 79, 80]. In Northern Sabah, Malaysia, village-level *P. knowlesi* incidence was positively associated with both forest cover and historical forest loss, with wider community exposure associated with forest fragmentation and agricultural practices [81, 82]. Deforestation is also associated with changes in macaque movements and increased contact between people and mosquito vectors at forest edges [83, 84].

Similarly, within the South American rainforests, a human infection with the simian malaria *P. simium* had been historically reported, although there was little evidence of widespread human infections until recently [53]. Since 1993, sporadic human cases of a *P. vivax*- like malaria infection were reported from the Atlantic forest region of Rio de Janeiro, Brazil, an area in which malaria had previously been eliminated. Parasitological and molecular investigations of these infections revealed human cases of *P. simium*, including 28 confirmed cases in 2015–2016 [85]. Naturally acquired human infections with the simian malaria *Plasmodium brasilianum* were confirmed in indigenous communities in the Venezuelan Amazon [86]. The increasing incidence and widespread circulation of these zoonotic malaria species poses significant threats to malaria eradication, highlighting the need to understand how risks evolve with future LULCC.

## Discussion

These rapidly changing landscapes have huge potential to undermine any future malaria eradication efforts. While increasing development, urbanization and expanded healthcare coverage are widely expected to reduce malaria risks globally [4], these trends also drive the increased needs for resources underlying most LULCC. Further, these changes exert increasing evolutionary pressures on ecological systems to adapt to changing environments. For example, while malaria is historically a predominantly rural disease in Africa, the urban malaria vector *An. stephensi* typically found in India has invaded areas of East Africa, largely driven by truck routes and trade [87, 88]. Malaria control and eradication strategies need to detect and adjust to changing epidemiological patterns. While LULCC impacts on socio-ecological systems driving malaria transmission are complex, priorities for malaria eradication strategies are outlined, highlighting the need for engagement across different sectors.

## Moving from global to local contexts: the importance of scale

One of the key lessons learnt from the previous malaria eradication failures is the need for context-specific national malaria elimination strategies with the flexibility to adjust to short and long term changes [4]. Highly effective control strategies in one context may be ineffective in other areas, for example, the limited utility of bed nets and indoor residual spraying in areas where transmission is driven by exophagic mosquito species and outdoor occupational activities [89]. A large volume of literature addresses this need to stratify approaches to malaria control and defines malaria “paradigms,” characteristics of ecosystems and populations relevant to control [90]. While this recognizes the heterogeneity of malaria transmission, higher levels of granularity in social and ecological factors are needed to accurately monitor and control malaria risks. For example, widely described “forest malaria” in Southeast Asia encompasses a range of transmission patterns, from hunting in deep forest environments to occupational risks at industrial rubber plantations to peri-domestic exposure around secondary forest edges near households [36, 83, 91]. These differences have critical implications for identifying populations at risk and effective interventions, requiring continued engagement of local control programmes and experts to design context-specific control measures.

Estimating the impacts of LULCC also requires understanding the wider socioeconomic and environmental contexts in which these changes occur. Primarily driven by economic forces, increased prosperity from LULCC can reduce malaria burdens despite ecological changes favourable to transmission [45]. Conversely, economic pressures driving LULCC can simultaneously weaken health systems and amplify ecological impacts. Within Venezuela, economic collapses and political instability have both crippled malaria control programmes and driven rapid deforestation due to migration to frontier areas for extractive activities [92, 93]. Changes to vector habitats and accompanying increases in vulnerability of human populations lead to a massive resurgence of malaria despite elimination of malaria within large regions of Venezuela in 1961 [94]. Venezuela now accounts for a substantial percentage of malaria within the Americas, threatening elimination and control programmes in surrounding countries [95]. Similarly, LULCC interacts with wider climate changes, either increasing or decreasing vulnerability to climate anomalies or longer-term changes.

Because of these interactions, associations between LULCC and malaria risks are modulated by the spatial and temporal scales of analysis. Initial LULCC impacts on disease transmission from disruption of existing ecosystems may change over time as transmission reaches new equilibrium states. Following deforestation, subsequent stages of forest succession and agricultural development may either create new habitats for disease vectors and hosts or lead to overall decreases in malaria burdens [7]. Ecological processes affecting the distribution of people, disease vectors and wildlife hosts may occur at highly local to larger regional scales [96]. For other vector-borne diseases, variations in host richness and ecological community structure have been shown to be important at a fine spatial scale while changes in climate and other abiotic factors are more important across larger scales [97].

## Linking health and environmental data for surveillance in changing landscapes

Monitoring these changes in malaria transmission requires detailed data on malaria infection and disease burden, human, mosquito and other host distributions and wider environmental factors collected in consistent ways across the relevant scales. The WHO now recognizes surveillance as a core intervention required to achieve malaria elimination. However, despite efforts to digitize and geolocate malaria surveillance data and advances in using climate data to inform malaria early warning systems [98], LULCC data rarely informs malaria surveillance.

**Fig. 4:**
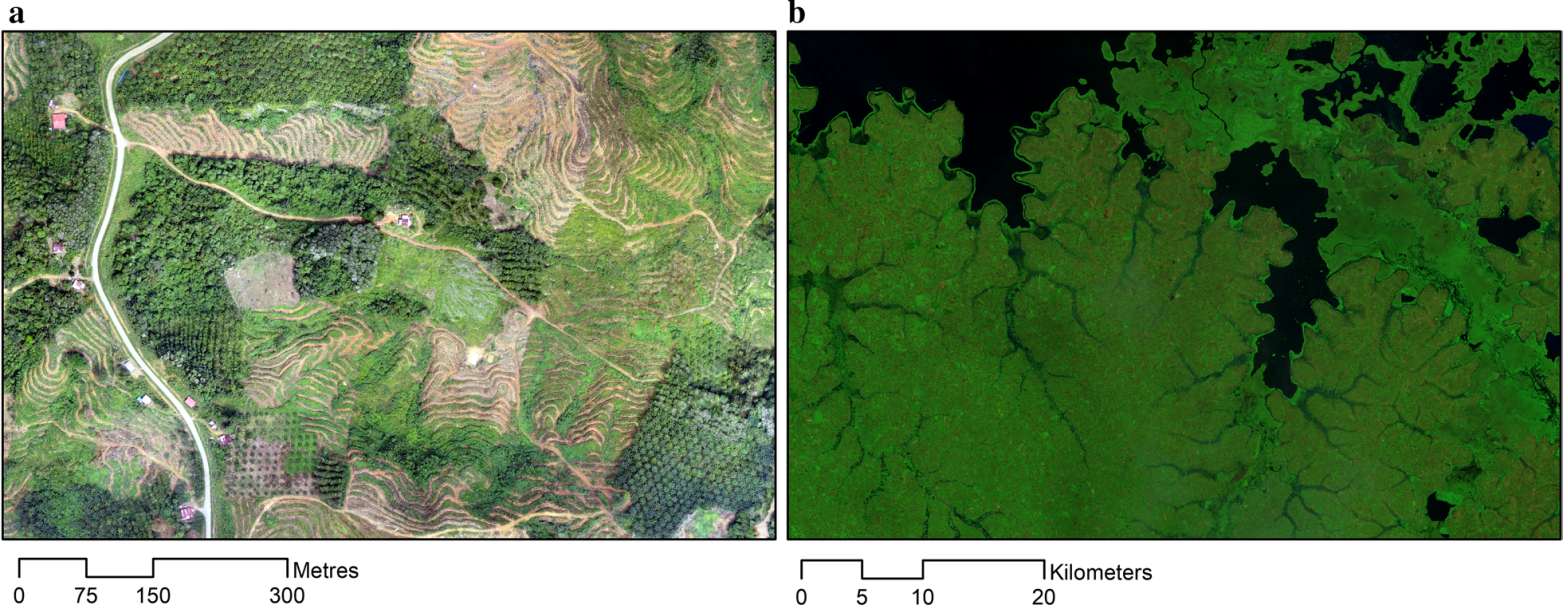
Examples of remote sensing data on landcover: a. very high-resolution data collected by UAV (11 cm per pixel) in Malaysian Borneo; b. false colour composite from LANDSAT satellite data of Lake Victoria in Uganda (30 m per pixel)

New sources of Earth Observation data offer unprecedented opportunities to detect changes in land cover and proactively target surveillance and control measures. Earth Observation data is widely used to monitor physical changes to the environment such as land cover and surface water changes; this data can be used to quantify extents of land cover changes as well as to characterize habitat configuration, such as levels of fragmentation and proximity of forests to households. High-resolution satellite imagery is freely available through governmental and international agencies such as NASA (https://eospso.nasa.gov/) and the European Space Agency (https://www.esa.int/ESA) with many countries additionally maintaining their own dedicated satellites. While health programmes can be limited by the technical, software and time required to process this data into a usable form, cloud-based computing platforms such as Earth on Amazon Web Services (https://aws.amazon.com/earth/) and Google Earth Engine (https://earthengine.google.com/) provide access to imagery and infrastructure to analyse planetary-level data. Additional online platforms, such as Global Forest Watch, publish processed data of forest cover and forest loss online in addition to near real-time deforestation mobile alerts designed to provide actionable information to government agencies [99]. Low-cost drones (unmanned aerial vehicles or UAVs) have also been utilized by malaria programmes in diverse ecological contexts including Malaysia, Tanzania and Peru [100–102]. Drones allow collection of fine-scale data at user-defined intervals and can be used to monitor deforestation, agriculture and development (Fig. 4). Despite the increasing accessibility of Earth Observation and spatial data, these are rarely used by health programmes and further work is needed to develop capacity to integrate these data within surveillance systems.

Malaria risk models have incorporated land use factors to develop spatially and/or temporal predictions of malaria risks, potentially allowing targeting of interventions and strategic planning [103]. Within research communities, datasets on land cover, land use and associated characteristics (such as vegetation indices or land surface temperatures) are widely used to identify areas with increased risk [104–108]. Data on landscapes and mosquito can be integrated with detailed behavioural and demographic risk determinants to explore plausible land use change scenarios and impacts on human health [109]. However, despite increasing use in scientific literature, there are fewer examples of LULCC data directly informing malaria surveillance programmes. Notably, Malaysia incorporates metrics of recent deforestation and recent construction activities into malaria foci investigations, defining receptivity based on numerous ecological and social factors [110]. More broadly, global planetary health projects have also highlighted the need to link both health and environmental data to monitor changing risks [111]. Major advances in computing, information technologies and environmental monitoring have tremendous potential to improve malaria surveillance and are a priority for future research and development.

## Building out malaria through sustainable development

Ultimately, achieving malaria eradication requires not only monitoring and responding to impacts of LULCC on malaria transmission but actively mitigating risks within future landscapes. Agriculture covers over 37% of global land surfaces, 50 million km^2^ globally [112]. These landscapes are entirely man-made, providing opportunities to design malaria resistant environments. Approaches to reduce malaria transmission within these landscapes generally comprise of three approaches: environmental modification on land, water or vegetation with long-lasting effects for vector habitat reduction; environmental manipulation that generates unfavourable temporary conditions for vectors; and modification of human habitation to reduce exposure to vectors.

A systematic review identified 16 studies that applied environmental modification and 8 studies that modified human habitation, reducing the risk ratio of malaria by 88% and 79.5%, respectively [113]. For example, cacao plantations under nurse trees in Trinidad generated ideal breeding sites within epiphytic bromeliads for *Anopheles bellator,* the main local malaria vector. Control of the resulting malaria epidemic was achieved through environmental manipulation with the modification of plantation techniques [28]. With the intent of preventing malaria epidemics, environmental manipulation has been proposed in Panama and other Latin American countries by increasing forest cover recovery in highly disturbed deforested areas, thus favouring the prevalence of auxiliary over primary vectors [34]. Malaria vector breeding sites can also be decreased through effective water management, mitigating potential effects of irrigation or dams. Utilization of intermittent irrigation in African rice fields has greatly reduced anopheline densities and increased rice yields while construction of several types of siphons and small dams in Sri Lanka and Malaysia’s rivers and streams eliminated mosquito breeding habitats. Environmental management interventions in the reservoirs of the Tennessee River Valley including an integrated operating rule for water fluctuation cycles, reduced *Anopheles* breeding sites significantly [113].

One of the most successful large-scale environmental modification interventions was during the construction of the Panama Canal. In 1878, this construction was halted due to engineering challenges, yellow fever and malaria and the resulting deaths amongst workers. Sanitation improvements allowed continuation of the project, including implementation of temporary and permanent drainage infrastructure and vegetation management, while dramatically decreasing malaria incidence [113]. More recently, plans for major developments have included evaluation of impacts on malaria transmission and preventive measures to mitigate these. For example, during the plans for Batu Hijau, a large-scale surface mine in Indonesia, environmental assessments highlighted impacts on community malaria risks, particularly in relation to lagoons and potential vector breeding sites. This prompted the establishment of a corporate public health programme focussing on environmental management, larvicides, mosquito control and active and passive detection and treatment of malaria cases [114]. Similarly, health programmes were incorporated into projects led by ExxonMobil in Papua New Guinea and hydroelectric projects in Lao PDR to address negative externalities of developments and present templates for future developments [114].

## Conclusions

The impacts of LULCC on malaria transmission are highly complex and context specific; environmental and demographic changes within a specific setting may lead to increases or decreases in malaria risks. Impacts may vary over space and time due to interactions between the environment and intrinsic factors such as species composition and ecology, demographic changes influencing socioeconomic status, risk behaviours and access to control measures. Malaria eradication will not be possible without accounting for these changing risks. This requires engaging with partners outside the health sector to develop interventions appropriate to local socio-ecological contexts, integrate environmental data into malaria surveillance systems and engineer malaria resistant landscapes.

## Data Availability

All data is publicly available. Full versions of World Health Organization reports and policies are available at 10.5281/zenodo.3753145 and https://www.who.int/publications/i/item/malaria-eradication-benefits-future-scenarios-feasibility.
